# Effects of Concentration and Surface Pressure on MBP Interaction with Cholesterol in Langmuir Films

**DOI:** 10.1155/2017/1542156

**Published:** 2017-11-07

**Authors:** Lei Zhang, Changchun Hao, Guoqing Xu, Runguang Sun

**Affiliations:** School of Physics and Information Technology, Shaanxi Normal University, Xi'an 710062, China

## Abstract

Predicting the mechanism of MBP binding to cholesterol is meaningful in understanding how MBP participate in lateral membrane organization. The interaction of MBP with cholesterol monolayer was investigated at three surface pressures on 10 mM Tris-HCl buffer with the different concentrations of MBP. The results show that *π*-*A* isotherms shift to larger molecular area at all pressures. By means of analyzing *π*-*T* curves, a surface pressure increase was obtained. Results indicated that the greater the protein concentration in the subphase, the larger the increase of surface pressure. In addition, changes in monolayer surface morphology and domain formation were performed by AFM. These results provide more direct and convincing evidence for the MBP interaction with cholesterol. The MBP-cholesterol interaction suggests a significant concentrations and surface pressure dependence and is probably governed by hydrogen bonds. The date presented could help to understand at least one of the molecular mechanisms through which MBP affects lateral organization of the cholesterol membrane.

## 1. Introduction

Cholesterol is an essential component of both central nervous system (CNS) and peripheral nervous system (PNS) myelin and acts as a precursor of signaling molecules in the CNS [1]. In addition, MBP is to be the main component in the maintenance and formation of integrity of CNS myelin. The interaction of proteins with lipid structures plays an important role in various fields [2]. A large number of biological reactions happened in interfaces where the main constituents are proteins and lipids. The systematic investigations of lipid-protein interactions have been performed using biomembrane models at the air-subphase interface of a Langmuir film balance. The Langmuir film balance is also known as Langmuir-Blodgett (LB) technology. It is used to study the relationship between the area and surface pressure at the liquid surface spread film. To date the LB technology is often coupled with AFM to determine the topography and domains of the monolayer at different subphase composition, surface pressure, pH, temperature, and so on [3].

Here, simple bioinformatics approaches were used to set up whether MBP, one of the major structural proteins of CNS myelin, is capable of adhesive cholesterol. MBP has been confirmed to be the main agent in the formation and maintenance of integrity of CNS myelin [4, 5]. According to the clinical experience and latest research, MBP was found to be associated with myelin degradation. Multiple Sclerosis (MS) attacks the myelin-wrapped nerves of the CNS [6]. MBP is water soluble protein with 170 amino acid residues and a molecular weight of 18.5 KDa with a net positive charge of 19 at physiological pH [7, 8]. Maintaining the myelin sheath that wraps around neurons by holding together both cytoplasmic sides of oligodendrocyte membranes is the primary physiological role of MBP [9]. In summary, MBP is an intrinsically unstructured (disordered) protein that may be combined to the polar lipid, such as phospholipids and cholesterol.

**Figure 1:**
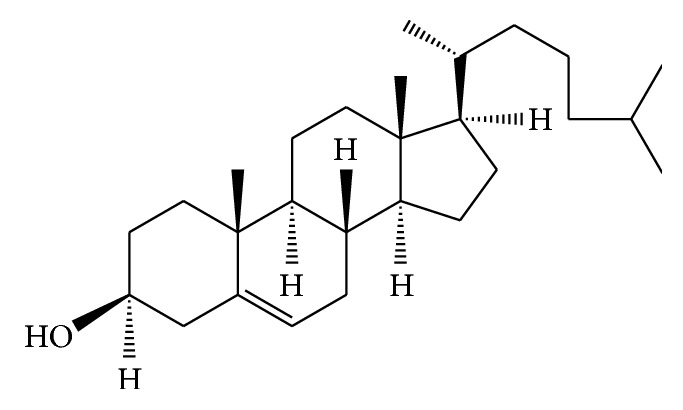
The chemical structure of cholesterol.

Notably, cholesterol is very special among biological membrane lipid because it is polycyclic, has a very small polar head group (-OH), and does not contain any acyl-chain that allows biochemical changes (Figure 1). At the same time, cholesterol is an indispensable and vital component of both PNS and CNS myelin, whose main role in the central nervous system is to act as a precursor of signaling molecules, such as oxysterols and neuroactive steroids [10, 11]. A variety of experimental data measured that the attraction and binding of MBP to monolayers or bilayers of different lipid compositions are modulated by hydrophobic interactions with the hydrophobic chains of lipids and electrostatic interactions with the acidic lipids head groups [12–15]. Recent evidence also manifests that the binding of MBP to cholesterol-containing membranes affects the formation of lipid microdomains [16]. Therefore, predicting which MBP could bind cholesterol is very important and meaningful in comprehending how MBP participate in lateral membrane organization of cholesterol. By applying physicochemical and chemoproteomic strategy, novel cholesterol-protein interactions in living cells have been recent describe [17].

Since cholesterol is a major component of myelin lipid membranes, the mechanism of their formation could incorporate interaction between MBP and cholesterol. Nevertheless, no interaction of cholesterol with MBP has been investigated in more detail. Here, LB technology was used to detect the adsorption of MBP to cholesterol surfaces as a function of surface pressure, adsorption time, and bulk MBP concentration.

The results obtained suggest that there is a possibility of such an interaction. These results of the research provide a new insight on at least one possible molecular mechanism of the integrity and functionality of cytoplasmic myelin monolayers.

## 2. Materials and Methods

### 2.1. Materials

MBP was extracted from bovine brain and purified in the water soluble according to established procedures of Deibler et al., [18] solubilized in Tris-HCl 10 mM, pH 7.2, and prepared in working solution at a concentration range of 1.0 × 10^−9^~5.5 × 10^−9^ M. Cholesterol (purity > 99%) was purchased in powder form from Avanti Polar Lipids, Inc. (Alabaster, AL, USA), and used without practicing further purification procedures. In the study of monolayers, cholesterol was dissolved in chloroform/methanol 3 : 1 (v/v) mixture at a final concentration of 1 mg/mL and used as spreading solution in general amounts of 20 *µ*L. All the experimental water (18.2 MΩcm) was acquired from a Millipore purification system.

### 2.2. Surface Pressure-Area Isotherms

All experiments were conducted on a KSV Mini-trough system (Helsinki, Finland) with an effective trough surface area of 75 × 364 mm^2^. The measurement resolution is ±0.1 mN/m with a trough volume of 240 mL.

The monolayer was spread by using a lipid solution on 10 mM Tris (hydroxyethyl) amino-methane titrated to pH 7.2 with HCl. The required volumes of the lipid solutions were deposited at the air/subphase interface containing the appropriate amount of MBP by using a Hamilton microsyringe. 15 minutes was left for the solvent evaporation after which the monolayers were compressed with the constant speed of 10 mm/min per barrier. The temperature of the subphase remains constant by means of the water circulator bath. Before each experiment, the trough and barriers were washed with absolute ethyl alcohol and rinsed with ultra-pure water.

Compression of the cholesterol monolayer occurred until the target surface pressure of 15 mN/m was reached. The surface pressure variation caused by the interaction of the MBP in the subphase with the cholesterol monolayer was continuously recorded as a function of time by using a computer-controlled LB until the surface pressure was in equilibrium, suggesting the end of adsorption. All measurement data were repeated three times minimum to ensure the reproducibility.

### 2.3. Atomic Force Microscopy (AFM) Observations

AFM image was acquired in air at room temperature using a SPM-9500-J3 AFM (Shimadzu Corporation, Japan). AFM images were obtained by using a Micro V-shaped Cantilever (Olympus Optical Co. Ltd., Japan) with a spring constant of 0.06 N/m, a length of 125 *μ*m, and thickness of 400 nm. All images (512 × 512 points) were performed in air at a scan rate of 1 Hz.

## 3. Results and Discussion

### 3.1. Thermodynamic Interaction of Monolayer Isotherms

**Figure 2:**
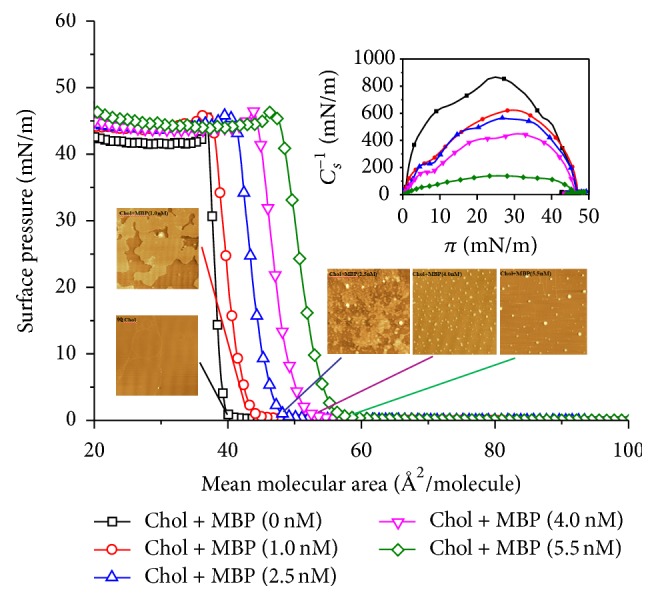
Surface pressure-area (*π*-*A*) isotherms and atomic force microscopy images of cholesterol monolayers on the Tris-HCl buffer containing different concentrations of MBP are shown (inset: variation of the compression modulus *C*_*s*_^−1^ with surface pressure *π*).

The surface pressure versus molecular area (*π*-*A*) isotherms of cholesterol monolayers spread on the buffer subphase containing MBP at different concentrations (0, 1.0, 2.5, 4.0, and 5.5 nM) were measured at 22 ± 1°C (in Figure 2). The resulting *π*-*A* curves evince the phase behavior of the monolayer in the course of compression, when the molecular packing gradually increases. For the isotherm of pure cholesterol monolayer, the curves are in agreement with those reported in the previous work [19–21]. As presented in Figure 2, with the increase of the MBP concentration in the subphase, the curves we studied were shifted towards the higher area. That is to say, they occupied a greater area at a given surface pressure than the membrane spread on pure water surface, implying that the presence of MBP induces minor changes of lipid conformations. Similar to our previous studies [22], when added to the subphase, MBP gave rise to the changes in the conformation of cholesterol monolayer. Furthermore, the changes of curves and surface conformation indicated that there were strong molecular interactions between the cholesterol and MBP. AFM observation of MBP/cholesterol shows domain at different surface pressures, and the monolayer becomes homogeneous promptly when the surface pressure is increased. The study found that the adsorption process mainly depends on the hydrogen bonds between hydroxylic groups of cholesterol and MBP, and MBP may also have a great effect on the conformation of cholesterol in the monolayers.

To quantify the phase transition process of the monolayers, we introduced the parameter of compression modulus (*C*_*s*_^−1^). The researchers have published that the presence of mixtures in lipid monolayers produced more compressible films which strongly depends on the size and hydrophobicity of lipid molecules [23]. It has become a significant parameter to characterize the transition region, based on the analysis of *π*-*A* isotherms. These experimental values were calculated by using the following equation:

(1) $$\begin{array}{c} C_{s}^{-1}=-A\left(\;\frac{d\pi }{dA}\;\right), \end{array}$$

where *A* is the area per molecule of the monolayer and *π* is the corresponding surface pressure [24]. *C*_*s*_^−1^ can be used to characterize the phase state of the monolayer (*C*_*s*_^−1^ = 12.5~50 mN/m, lipid expanded, *C*_*s*_^−1^ = 100~250 mN/m, lipid condensed) [25]. Another role is used to compare the elastic modulus of different monolayer. The higher *C*_*s*_^−1^ value the more ordered the monolayers [26].

**Figure 3:**
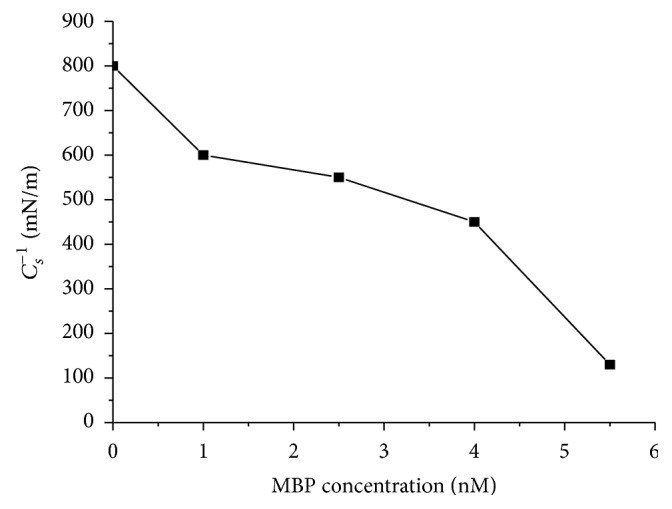
Plot of compression modulus (*C*_*s*_^−1^) as a function of MBP concentration in the subphase.

As presented in the inset to Figure 2, we show the changes of *C*_*s*_^−1^ with *π* for the monolayers of MBP/cholesterol. For the monolayer of pure cholesterol, it presents a typical condensed monolayer with a maximum value of *C*_*s*_^−1^ above 800 mN/m (solid phase). Cholesterol monolayer formed on water subphases has been studied in various works [27]. In our case, the compression modulus *C*_*s*_^−1^ of the monolayers mixtures MBP/cholesterol dropped with an increase in MBP concentration (Figure 3). It can be concluded that the incorporation of MBP into the monolayers of cholesterol causes a decrease in *C*_*s*_^−1^ value, and the monolayers become more disordered. This behavior indicated that MBP interacts with the cholesterol molecules in air/subphase interface.

### 3.2. Quantitative Analysis of Successive Compression-Expansion Cycles

**Figure 4:**
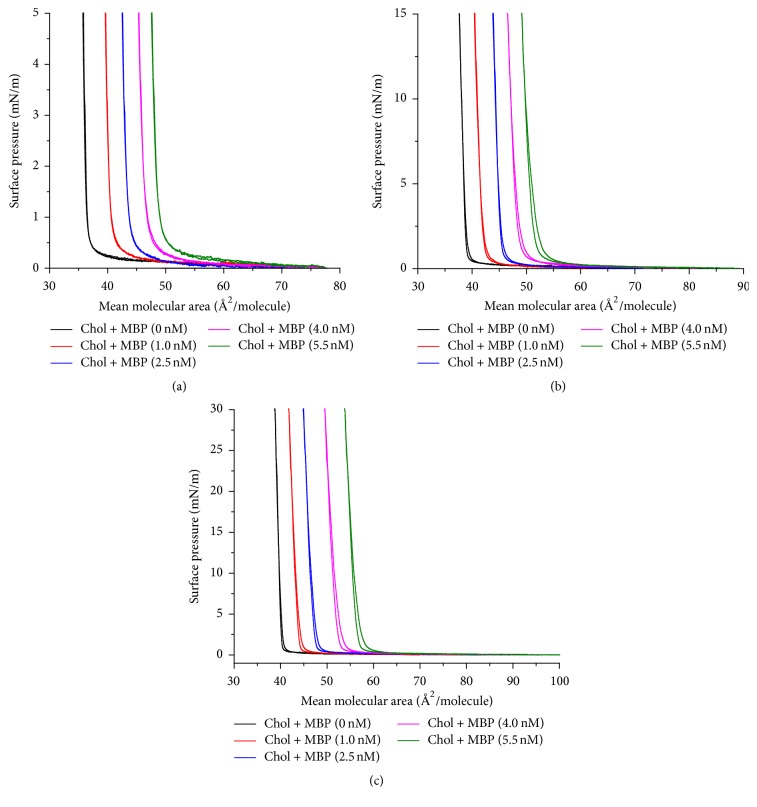
Compression-expansion cycles of cholesterol on the different concentrations of MBP at variable surface pressure values.

In order to investigate the stability of mixed monolayer successive compression-expansion cycles in air/subphase interface were conducted (see Figures 4(a), 4(b), and 4(c)). The results of consecutive hysteresis cycles of cholesterol monolayers over the different concentrations of MBP at variable surface pressure values were shown in Figure 4. The *π*-*A* isotherms of MBP/cholesterol mixed monolayer shift entirely to higher areas which indicates that the interaction between MBP and cholesterol is strongly influenced by the concentration of MBP in the subphase. By analyzing the compression-expansion cycles of three surface pressures (5, 15, and 30 mN/m), we founded that the expansion isotherm moves to the left slightly relative to that of the compression one. The reason for this phenomenon suggested that MBP/cholesterol mixed monolayer shows a mild hysteresis behavior with almost no loss of material into the Tris-HCl subphase (Figure 4). At the same time, pure cholesterol does not show hysteresis. It is shown in Figure 4 that all cholesterol/MBP films demonstrate some degree of hysteresis after at different concentrations of MBP adsorption, where the compression curves of the films happen to at higher molecular areas than the expansion part of the curves. The quantification of the compress-expansion cycle of apparent losses in the terms of % is defined according to

(2) $$\begin{array}{c} Apparent\;\;loss\left(\;\%\;\right)=100\left(\;1-\frac{A_{E1}}{A_{C1}}\;\right). \end{array}$$

Wherein *A*_*C*1_ and *A*_*E*1_ are the values of molecular area of the first compression curves for the different concentrations and surface pressures, respectively. The value of apparent losses is used to describe the effects of the various parameters such as the surface pressure and the concentration of protein in the subphase. The numerical characteristics of the apparent loss are presented in Table 1.

The data in Table 1 we analyzed show that the greater the concentration of MBP and the surface pressure, the more the value of apparent loss; however, we noticed that the apparent loss of hysteresis is very minor at the three surface pressures. The maximum value of 3.34% of apparent loss is acquired, suggesting small changes of mixed monolayer at the MBP concentration of 5.5 nM and the surface pressure of 30 mN/m. This result indicated more stable interaction between MBP and cholesterol in this phase.

### 3.3. Affinity Capacity of MBP onto a Cholesterol Monolayer

**Figure 5:**
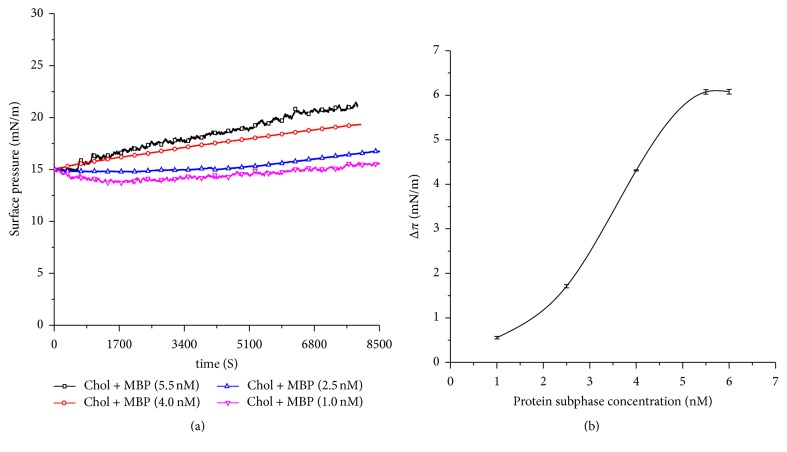
(a) Time dependence of the surface pressure changes during the adsorption of MBP with the cholesterol monolayer. *C*_MBP_ = 1.0, 2.5, 4.0, and 5.5 nM. (b) Surface pressure increase (Δ*π*) of a cholesterol monolayer (*π*_initial_ = 15 mN/m) induced by different subphase concentrations of MBP.

The affinity ability of MBP onto a cholesterol monolayer was conducted by LB experiments at various concentrations of the MBP in the subphase.  Figure 5(a) shows the increase in surface pressure (Δ*π* = *π*_final_ − *π*_initial_), as the cholesterol is spread over the interface with an initial surface pressure of 15 mN/m. The increase of surface pressure implied MBP insertion into the cholesterol monolayer for different MBP concentrations. With the increase of the MBP concentration in the subphase, the increase of the surface pressure also increases (Δ*π*_5.5 nM_ > Δ*π*_4.0 nM_ > Δ*π*_2.5 nM_ > Δ*π*_1.0 nM_). A plateau is observed after 6800 s, which demonstrates that the MBP adsorb to the cholesterol film processes at the lipid interface or in the bulk solution (aggregation).  Figure 5(b) should stress on the fact that the ability of MBP adsorbing to monolayer largely depends on the concentration of MBP and the composition of monolayer at the air-subphase interface.

### 3.4. Surface Morphology Explores by Atomic Force Microscopy

**Figure 6:**
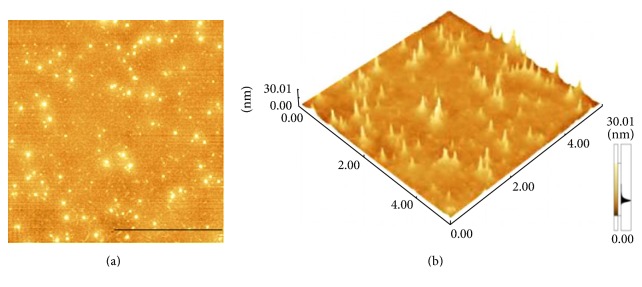
MBP molecules by Langmuir-Blodgett deposition. (a) is the height map of (b). Scale bar: 5 *µ*m.

**Figure 7:**
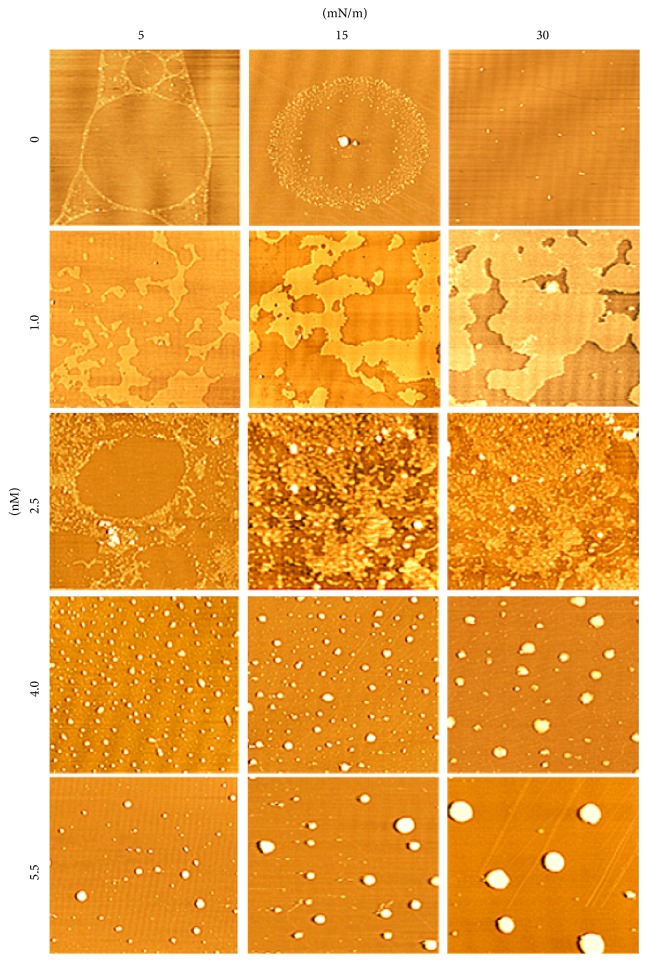
AFM image of the binary MBP/cholesterol monolayers at three surface pressure (5, 15, and 30 mN/m) on 10 mM Tris-HCL buffer (pH 7.2) at *C*_MBP_ = 0, 1.0, 2.5, 4.0, and 5.5 nM. The scale bars in the lower-right represent 5 *µ*m.

The monolayers at the air/subphase interface were transferred onto smooth mica substrates for detection by AFM. Our main goal was to detect whether MBP would cause conformational changes in the formation of the cholesterol monolayer. The initial surface pressure of the lipid monolayers was set at 5.0 ± 1.0, 15.0 ± 1.0, and 30.0 ± 1.0 mN/m and the MBP concentrations in the subphase were 0, 1.0, 2.5, 4.0, and 5.5 nM, respectively. In Figures 6(a) and 6(b), MBP prepared by Langmuir-Blodgett method have been visualized by AFM. These granules have been evidently formed by MBP molecules. Changes in the surface morphology of the cholesterol monolayer induced by the interaction with MBP were reflected with AFM.  Figure 7 shows that the morphological images of the mixed monolayer vary significantly with concentration of MBP and surface pressure. Figures 7(a), 7(b), and 7(c) display the AFM images of pure cholesterol monolayers deposited at 5, 15, and 30 mN/m, respectively. There has been a single phase structure with the line tension forming a pattern of circular domain at the lateral pressure of 5 mN/m and finally showing a homogeneous and dense phase at the surface pressure of 30 mN/m.

**Figure 8:**
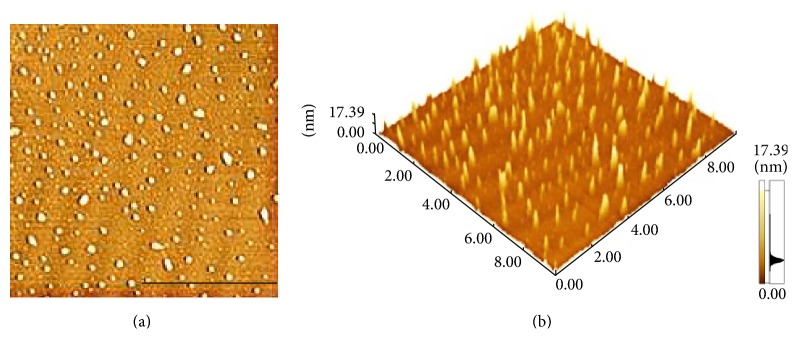
AFM image of MBP adsorption into spread cholesterol monolayer from the Tris-HCl subphase at the concentration of 4 nM. (a) is the height map of (b). Scale bar: 5 *µ*m.

**Figure 9:**
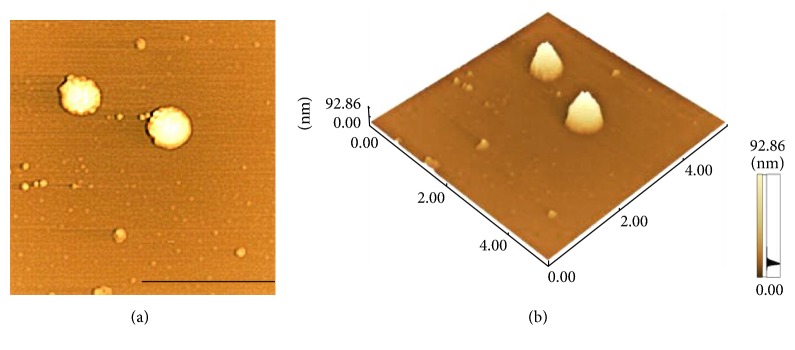
AFM image of MBP adsorption into spread cholesterol monolayer from the Tris-HCl subphase at the concentration of 5.5 nM. (a) is the height map of (b). Scale bar: 2.5 *µ*m.

The presence of MBP in the subphase could induce changes dramatically to the topographic view of the monolayer. At the MBP concentration in the subphase of 1 nM, randomly distributing small irregular sheet structures can be found in the domains (Figures 7(d), 7(e), and 7(f)). The film is getting more density when the surface pressure reached 30 mN/m. The image difference in the adsorption surface pressure, albeit small, is meaningful. From the AFM image, it can be inferred that the sheet structures of mix monolayer differed from that of the pure cholesterol monolayer, indicating that incorporation of MBP in cholesterol monolayers disturbs the cholesterol organization. We also have presented the image for mixed monolayer at the MBP concentration in the subphase of 2.5 nM (Figures 7(g), 7(h), and 7(i)). As can be observed at the lateral pressure of 15 mN/m, MBP adsorption in the monolayer was randomly distributed, and some MBP molecular was aggregated with each other. As the MBP concentration increases to 4 nM, the scattered protein particles were observed, most of which formed the approximate shape of a spherical cap (Figures 7(j), 7(k), and 7(l)). We can analyze that these MBP molecules seem to aggregate and increase in size, as shown in Figure 6. At higher subphase MBP concentration of 5.5 nM, these MBP molecular aggregates appeared to be linked together to form larger structure (Figures 7(m), 7(n), and 7(o)). And you can see that in the height diagram (Figures 8 and 9).

This result supports the *π*-*A* isotherms and hysteresis behaviors that demonstrated that the morphological changes of the cholesterol monolayer induced by the interaction with MBP. As hydrogen bond formations are considered to be involved in the interaction of MBP to cholesterol membranes.

## 4. Conclusions

Our research provides experimental evidence for an interaction between MBP and cholesterol. The surface behavior of MBP with cholesterol was systematically investigated by applying the Langmuir-Blodgett technique. The monolayers of the cholesterol were spread on the subphase containing different concentrations of MBP, and their curves were measured. The important findings of this work can be summarized as the following three aspects. First of all, the *π*-*A* and hysteresis isotherms reveal that the MBP binding to the cholesterol is adsorbed to the surface of the monolayer. Secondly, the experimental results of the AFM image obtained by the present study demonstrated the conformational changes of the cholesterol monolayer induced by the different concentrations of MBP in the subphase. In the meantime, the formation of MBP aggregates through interaction between the hydroxyl groups of cholesterol and MBP by hydrogen bonding. And lastly, the concentration of MBP in the subphase is major factor influencing the adsorption of MBP in addition to cholesterol-MBP hydrogen bonding including hydrophobic cholesterol-MBP interactions. Hence, the results of the study provide particulars to understanding of the interaction of MBP-cholesterol on the molecular mechanism. They may provide useful and meaningful in CNS and PNS as a precursor of signaling molecules.

## Figures and Tables

**Table 1:** The apparent loss of the monolayers at the different subphase concentrations and the different surface pressure.

*π* (mN/m)	apparent loss%				
	0 nM	1.0 nM	2.5 nM	4.0 nM	5.5 nM
5.00	0.20%	0.30%	0.10%	0.09%	0.11%
15.00	0.15%	0.90%	1.00%	1.93%	3.12%
30.00	0.21%	1.95%	2.01%	2.63%	3.34%
